# Untargeted Proteomics-Based Approach to Investigate Unintended Changes in Genetically Modified Maize for Environmental Risk Assessment Purpose

**DOI:** 10.3389/ftox.2021.655968

**Published:** 2021-06-22

**Authors:** Sarah Zanon Agapito-Tenfen, Miguel Pedro Guerra, Rubens Onofre Nodari, Odd-Gunnar Wikmark

**Affiliations:** ^1^GenØk Centre for Biosafety, Tromsø, Norway; ^2^CropScience Department, Federal University of Santa Catarina, Florianópolis, Brazil

**Keywords:** transgenic organisms, 2-D DIGE, profiling techniques, maize, allergenicity assessment

## Abstract

Profiling technologies, such as proteomics, allow the simultaneous measurement and comparison of thousands of plant components without prior knowledge of their identity. The combination of these non-targeted methods facilitates a more comprehensive approach than targeted methods and thus provides additional opportunities to identify genotypic changes resulting from genetic modification, including new allergens or toxins. The purpose of this study was to investigate unintended changes in GM Bt maize grown in South Africa. In the present study, we used bi-dimensional gel electrophoresis based on fluorescence staining, coupled with mass spectrometry in order to compare the proteome of the field-grown transgenic hybrid (MON810) and its near-isogenic counterpart. Proteomic data showed that energy metabolism and redox homeostasis were unequally modulated in GM Bt and non-GM maize variety samples. In addition, a potential allergenic protein—pathogenesis related protein −1 has been identified in our sample set. Our data shows that the GM variety is not substantially equivalent to its non-transgenic near-isogenic variety and further studies should be conducted in order to address the biological relevance and the potential risks of such changes. These finding highlight the suitability of unbiased profiling approaches to complement current GMO risk assessment practices worldwide.

## Introduction

Genetically modified organisms (GMOs) have been extensively grown and consumed in a number of countries since 1998. Twenty-years after the first cultivation, the accumulated genetically modified (GM) crop area surged to a record of 191.7 million hectares in 26 countries around the world (ISAAA, 2017). Despite the widespread use of GMOs, the need for biosafety science remains a concern and it is mandated in the domestic legislation of many countries as well as in international treaties (Davison, 2010; Eckerstorfer et al., 2019).

Confidence in the safety and reliability of GMO food products depends significantly on the genetic integrity of the organism; however, the frequency of transformation-induced mutations which could result in altered metabolism, novel fusion proteins, or other pleiotropic effects leading to adverse effects are poorly understood (Zolla et al., 2008; Brandão et al., 2010; Kohli et al., 2010; Agapito-Tenfen et al., 2013). In fact, the transgene insertion site cannot be predetermined and for this reason transgenes may be inserted in functional genomic regions thus disrupting the structure and/or altering the regulation patterns of genes from the plant host genome as previously observed for some commercialized GM crops (Holck et al., 2002; Hernández et al., 2003; Rosati et al., 2008; Morisset et al., 2009; La Paz et al., 2010). Other secondary unintended effects of genetic modification can also arise during conventional breeding as the result of hybridization or spontaneous mutations, processes that are integral to breeding programs (van Gelder and Scheffer, 1991; Conner and Jacobs, 1999; FAO/WHO, 2002). Another documented effect is related to the application of supporting technologies used in the GMO agroecosystem, such as the use of combined herbicides (Bøhn and Millstone, 2019).

Profiling technologies, such as proteomics, allow the simultaneous measurement and comparison of thousands of plant components without prior knowledge of their identity. The combination of these non-targeted methods facilitates a more comprehensive approach than targeted methods and thus provides additional opportunities to identify genotypic changes resulting from genetic modification, including new allergens or toxins (Ruebelt et al., 2006; Agapito-Tenfen et al., 2013). The identification of such changes in the GMO that could cause adverse effects on the conservation and sustainable use of biological diversity, taking also into account risks to human health, is a first step in the GMO risk assessment process (United Nations Environment Programme, 2016).

Two-dimensional electrophoresis (2-DE) gel-based proteomic approaches have been widely used to investigate the protein-level metabolism of transgenic maize, soybean, cotton, rapeseed and rice in contrast to their non-transgenic counterpart in the past decade (Ren et al., 2009; Kun et al., 2010; Coll et al., 2011; Barbosa et al., 2012; Liu et al., 2015; Wang et al., 2015; Benevenuto et al., 2017; Galazzi et al., 2019). However, these studies do not report consistent results, which may be explained by their use of a variety of different genetic backgrounds and/or different growth conditions, as well as variations in the technologies applied (Ricroch et al., 2011). These inconsistencies highlight the importance of building a “database” of knowledge around genetic variability in GM crops, as well as the need for harmonization of analytical methods that could be addressed through continuous multi-laboratory tasks (Batista and Oliveira, 2010; Zanatta et al., 2020).

Among the different omics platforms investigating the proteome, 2-DE gel-based approaches enable the identification of protein isoforms that would not be possible by means of high throughput omics systems. In the present study, we used bi-dimensional gel electrophoresis based on fluorescence staining, coupled with mass spectrometry in order to compare the proteome of the field-grown transgenic hybrid (MON810) and its near-isogenic counterpart commercially available in South Africa. Protein profiles were generated and compared between the two plant varieties to assess differences in protein expression. Differentially expressed proteins were successfully identified and their molecular function and cellular components were analyzed. We observed imbalanced redox metabolism and a potential allergenic protein in GM maize expressing Bt toxin which have been grown in field conditions mimicking real world agricultural scenarios.

## Materials and Methods

### Plant Material and Growing Conditions

The cultivation of GM maize MON810 event (unique identifier MON-ØØ81Ø-6, Monsanto Company), also known as Bt-maize, has been approved in South Africa in 1997 (CERA, 2012). MON810 was genetically modified by particle bombardment to genomic insert the modified *cry1Ab* gene from *Bacillus thuringiensis*. The expression product of this gene is the insecticide protein (Bt toxin) Cry1Ab. White maize variety PAN 6Q-321B containing MON810 event (Pannar Seed Ltda., South Africa) and its non-GM near isogenic variety PAN 6Q-121 (Pannar Seed Ltda., South Africa) were planted in November 2009. These are single-cross hybrid seeds which are the progeny derived from the cross of a maternal endogamous line “A” with the paternal endogamous line “B.” This seed population is, therefore, highly genetically similar (all genotype should be AB).

After the confirmation of MON810 event in GM seeds and the absence in its near isogenic non-transgenic (non-GM) counterpart (data not shown), plants were grown side by side in 2.4 Ha blocks (density of 20,000 plants/Ha) in the same field located at the University of Free State Research Farm, Bloemfontein, South Africa. Plots were managed following standard agricultural practices in the region, without the application of herbicides. No fungicide or insecticide was either applied.

Six plants were randomly sampled per maize hybrid from each plot inner rows, in order to avoid border effects. Maize leaves were collected at R1 stage (~90 days after sowing). Sampling was performed during early morning in which around 5 g of material was collected from the third upper leaf, consisting of a 10 cm long tissue piece located in the mid portion. Plant samples were carefully checked for the absence of herbivory and disease symptoms, as well as necrotic tissue areas. The leaves were cut, placed in 15 ml tubes before immersion in liquid nitrogen and transported to the lab. The samples were kept at −80°C until used.

### Protein Extraction and Sample Labeling for 2-D DIGE Gel Electrophoresis

Each sample was separately ground-up in a mortar with liquid nitrogen and protein extraction was subsequently carried out according to Carpentier et al. (2005) with some modification. Phenol extraction and subsequent methanol/ammonium acetate precipitation was performed and PMSF was used as protease inhibitor. Pellets were re-suspended in an urea/thiourea buffer compatible to DIGE (4% w/v CHAPS, 5 mM PMSF, 7 M urea, 2 M thiourea and 30 mM Tris base; all reagents were purchased from Sigma-Aldrich Corporation, St. Louis, USA). Protein quantification was determined by means of the copper-based method 2-D Quant Kit (GE Healthcare Bio-Sciences AB, Uppsala, Sweden). A pool of 60 μg of protein samples per variety (consisting of equal amounts of each of the six plants assessed per treatment) were labeled with 400 ρmol/μl of CyDye DIGE fluors (GE Healthcare Bio-Sciences AB, Uppsala, Sweden), according to the manufacturer's instructions. Each pool was first separately labeled with a different fluor. After protein-fluor hybridization, samples were treated with lysine (10 mM) to stop the reaction and then mixed together for 2-D DIGE gel electrophoresis separation.

### 2-D DIGE Gel Electrophoresis Conditions

In order to determine the biological variance among our samples, a preliminary test has been carried out to established baseline variation information on samples collected for this study (Coll et al., 2011). The pre-test consisted of 450 μg of each of the six unlabeled samples from each variety which were then separated by 2-D gels using Immobiline™ DryStrip gels of 13 cm and a linear pH range of 4–7 (GE Healthcare) coomassie brilliant blue G-250 colloidal stained gels (Candiano et al., 2004). 2-D gel electrophoresis conditions were performed as described by Weiss and Gorg (2008).

Once determined that variability within samples were minimal and fell within the optimal range for proteomic analysis, the extracted proteins were separated by two-dimensional gel electrophoresis (Weiss and Gorg, 2008). In the isoelectric focusing (IEF) step, strip gels of 24 cm and a linear pH range of 4–7 (GE Healthcare) were used. Strips were initially rehydrated with labeled protein samples and a rehydration solution [7 M urea, 2 M thiourea, 2% w/v CHAPS, 0.5% v/v IPG buffer (GE Healthcare), 0.002% w/v bromophenol blue]. Strips were then processed using an Ettan IPGPhor IEF system (GE Healthcare) in a total of 35,000 Volts.h^−1^ and subsequently reduced and alkylated for 30 min under slow agitation in a Tris-HCl solution (75 mM), pH 8.8, containing 2% w/v SDS, 29.3% v/v glycerol, 6 M urea, 1% w/v dtt and 2.5% w/v iodocetamide. Strips were placed on top of SDS-PAGE gels (12%, homogeneous) and used in the second dimension run with a Hoefer DALT system (GE Healthcare). Gels were immediately scanned with the FLA-9000 modular image scanner (Fujifilm Lifescience, Dusseldorf, Germany).

Preparative gels for each treatment were also performed in order to extract spots with statistical significance differential expression between varieties. These were performed with a 700 μg load of total protein pools in 24 cm gels from each treatment, separately, and stained with coomassie (MS compatible) (Candiano et al., 2004).

### Gel Analysis

For the purpose of addressing plant-to-plant variability within our GM and non-GM varieties, the pre-test experiment consisted of 12 gels, six from each variety. These were analyzed all together by software Image Master 2D Platinum, version 7.0 (GE Healthcare). Gels were compared and matched spots volume of each gel was used to determine the biological variation by Principal Component Analysis (PCA) using Euclidean distance for quantitative analysis. PCA was first applied to determine the proportion of the total proteomic variation that originates from differences between biological repetitions. PCA analysis was performed by examining similarities of correlations between the observed measures. The analysis was carried out using covariance matrix performed by Multibase PCA PLS Cluster-Analysis Excel Add-in Program (Numerical Dynamics, 2013). The 2-DIGE experiment consisted of four technical replicate gels, each containing a loading pool of six biological replicates per variety. Cross-comparisons among the different samples were performed using software Image Master 2D Platinum, version 7.0. Hierarchical matching of gels was organized in such a way that technical replicate gels were compared fist and exclusive spots were removed from subsequent analysis. To analyze gel similarities or experimental variations, such as disparities in stain intensity or sample loading, scatter plots based on a linear dependence between the spot values of one gel and the corresponding values in the reference gel were produced. Spots within each variety with a high coefficient of variance (>20%) were excluded from the analysis. Therefore, only consistent spots for each variety were used in the comparative analysis. Statistical analyses were performed with the Student's *t* test (95% confidence interval).

### In-gel Digestion and Protein Identification by MS/MS

Gel spots were excised and subjected to *in-gel* reduction, alkylation, and tryptic digestion using 2–10 ng/μl trypsin (V511A; Promega) (Shevchenko et al., 1996). These were analyzed by the Proteomics Platform at the Arctic University of Norway (UiT). Peptide mixtures containing 0.1% formic acid were loaded onto a nano ACQUITY Ultra Performance LC System (Waters Massachusetts, USA), containing a 5-μm Symmetry C18 Trap column (180 μm × 20 mm; Waters) in front of a 1.7-μm BEH130 C18 analytical column (100 μm × 100 mm; Waters). Peptides were separated with a gradient of 5–95% acetonitrile, 0.1% formic acid, with a flow of 0.4 μl/min eluted to a Q-TOF Ultima mass spectrometer (Micromass; Waters). The samples were run in data dependent tandem MS mode. Peak lists were generated from MS/MS by the Protein Lynx Global server software (version 2.2; Waters). The resulting pkl files were searched against the NCBInr 2011120 protein sequence databases using Mascot MS/MS ion search (Matrix Sciences; http://matrixscience.com). The taxonomy used was Viridiplantae (Green Plants) and “all entries” for contamination verification.

**Figure 1:**
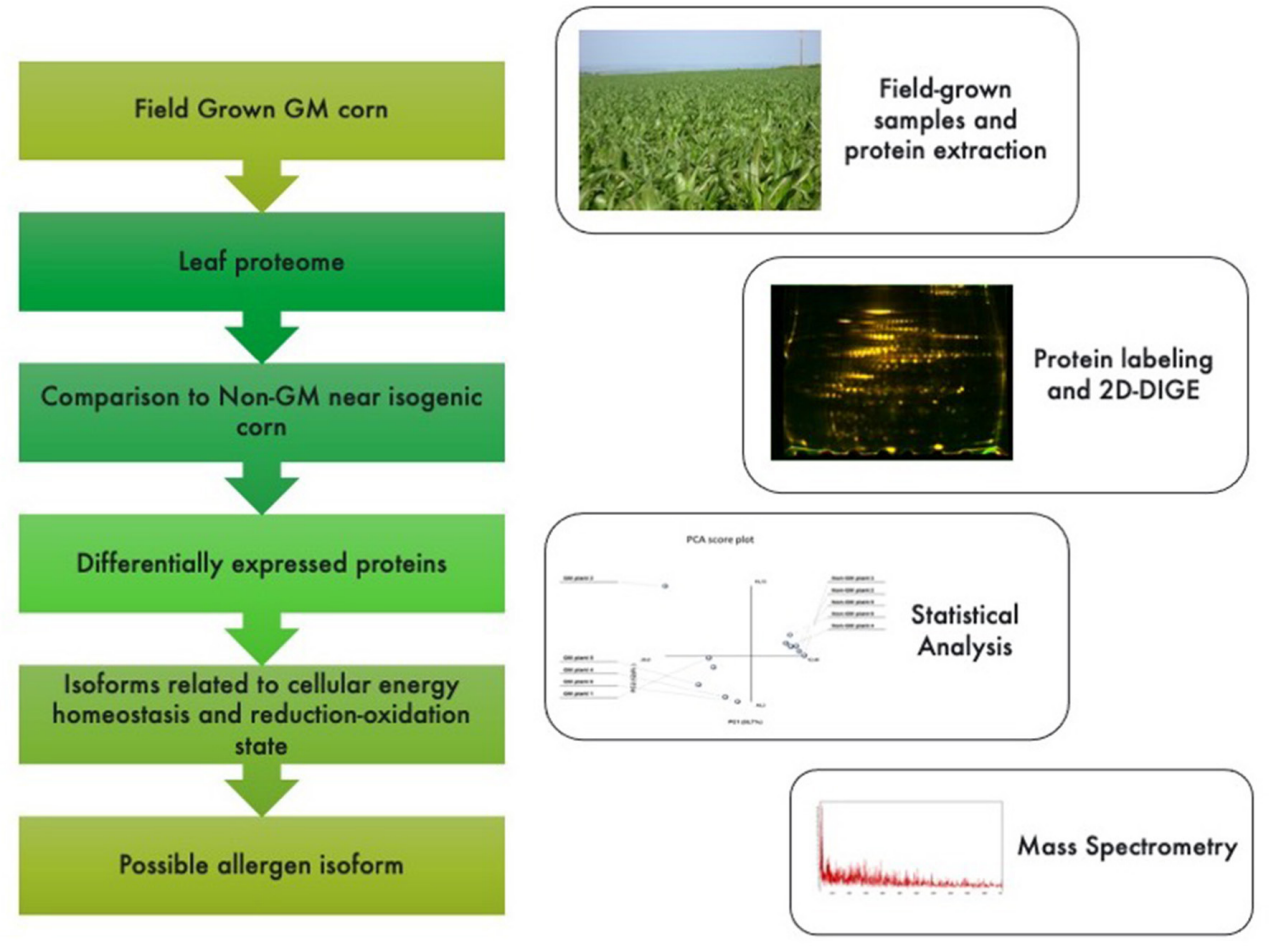
Graphical abstract and methodological pipeline for this study. Proteomic profiling analysis was performed for GM vs. non-GM maize samples expressing the Cry1Ab cassette. Plants were field grown in South Africa and subjected to a phenol-based protein extraction. 2-D fluorescent gels were analyzed and statistically significant spots (<0.05%) were sequenced by MSMS analysis. Identified proteins were then searched against public databases for their annotations.

The following parameters were adopted for database searches: complete carbamidomethylation of cysteines and partial oxidation of methionines; peptide mass tolerance ±100 ppm; fragment mass tolerance ±0.1 Da; missed cleavages 1; and significance threshold level (*P* < 0.05) for Mascot scores [−10 Log(*P*)]. Even though high Mascot scores are obtained with significant values, a combination of automated database searches and manual interpretation of peptide fragmentation spectra were used to validate protein assignments. Molecular functions and cellular components of proteins were compared against ExPASy Bioinformatics Resource Portal (Swiss Institute for Bioinformatics; http://expasy.org) and Gene Ontology Consortium (http://geneontology.org). Genome location for each protein was searched against Maize Genome Sequencing Project (http://www.maizesequence.org/index.html) by using the protein name. A database search for allergenic epitopes was performed at the Allergen Database for Food Safety (ADFS; Division of Biochemistry and Immunochemistry of National Institute of Health Sciences; http://allergen.nihs.go.jp/ADFS/). A graphical abstract is presented in Figure 1.

## Results and Discussion

### Suitability and Reproducibility of 2-D Gel-Based DIGE Experiments

Profiling techniques are broadly accepted as being capable of delivering sound descriptions of their target class of molecule in a range of diverse field from molecular medicine to food safety and plant physiology studies (Karahalil, 2016; Argueso et al., 2019; Mehta et al., 2019; Carrera et al., 2020). A number of molecular profiling studies have already indicated unintended effects of genetic modification (Coll et al., 2011; Wang et al., 2015, 2019; Zanatta et al., 2020). These studies revealed *inter alia* that compared to an effect of the genetic modification in the GM plants, there is also effects on the plant's physiology arising from (i) the genetic background, (ii) environmental conditions during growth, (iii) sampling procedures and (iv) plant-to-plant variability. Even the growth condition of the previous generation (such as the production of seeds) is known to cause epigenetic effects (Zolla et al., 2008).

Gel-free high-throughput mass spectrometry (MS) approaches have been applied in the past years to identify proteins on a larger scale with higher sensitivity compared to the traditional compositional analysis and reveal new aspects of the protein-level regulatory metabolism of GM crops (García-Cañas et al., 2011; Anguraj Vadivel et al., 2015). However, 2-DE technology is irreplaceable because it yields visualization maps of protein profiles, which provide information on the abundance of proteins and reliable evidence for existing protein isoforms (Benešová et al., 2012; Fonseca et al., 2012; Tan et al., 2017). Therefore, two-dimensional gel electrophoresis (2DE) is still one of the most important techniques, mostly due to its high performance regarding the separation of complex mixtures of full-length proteins. Ultimately, gel-free and gel-based approaches are both of great value to a proteomic study and often provide complementary information for an overall richer analysis (Abdallah et al., 2012). Comparative proteomic analysis requires reliable methods for investigation of differential protein expression. An important source of variation is derived from technical artifacts or heterogeneities (i.e., differences between sample collection, IEFs, gel runs). Blocking enables an effective comparison between observed conditions with little dependence on technical heterogeneities thus improving the precision of the statistical analyses (Valledor and Jorrín, 2011). This approach has been successfully applied to reduce bias related to protein labeling in 2-D DIGE experiments.

**Figure 2:**
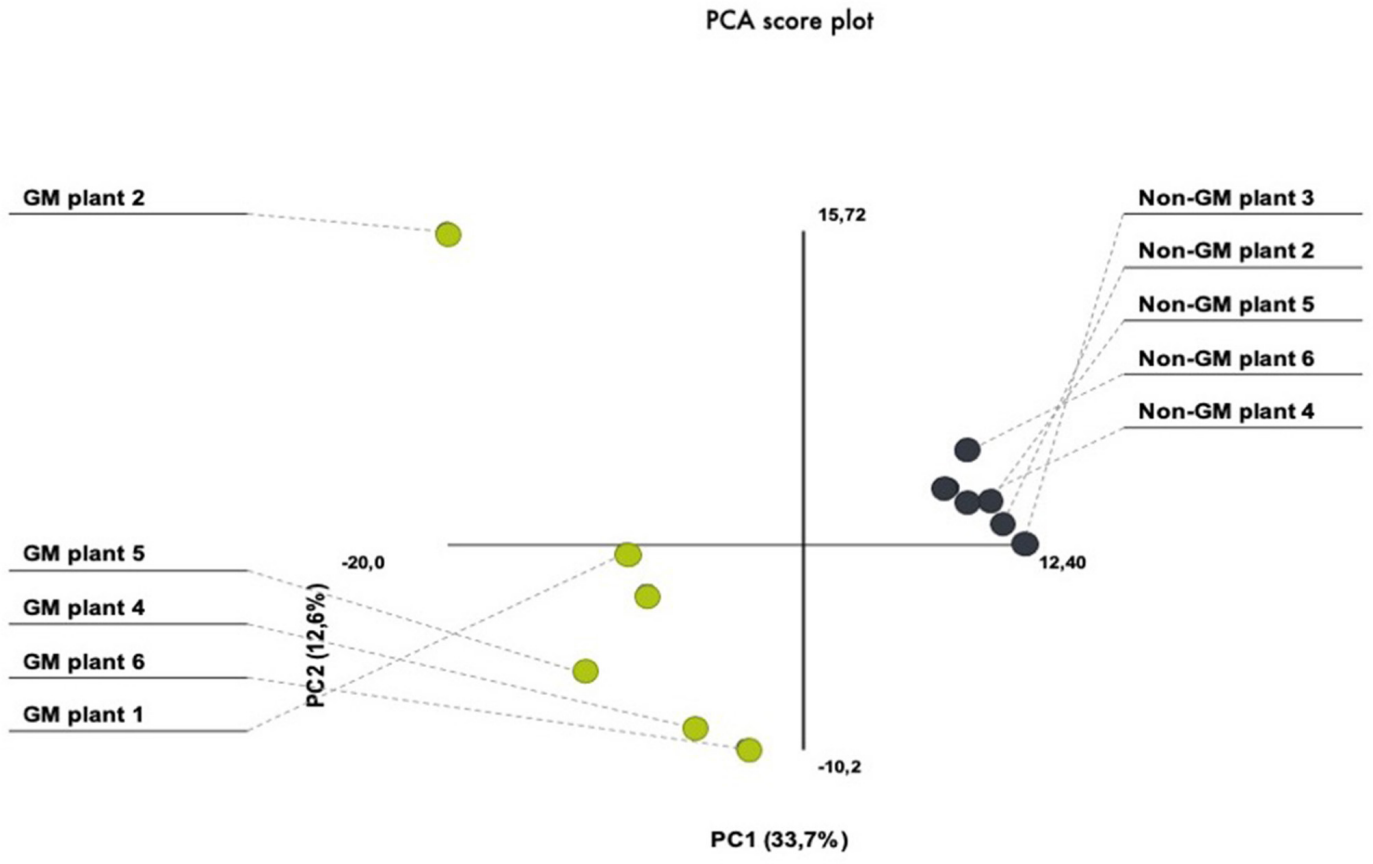
PCA score plots of 2-D proteomic data from transgenic (MON810 event) and non-transgenic near-isogenic maize plants (PAN 6Q-121) grown side-by-side under agricultural conditions at Bloemfontein, South Africa.

In the present investigation, we have chosen the nearest isogenic counterpart as the appropriate comparator. This is in agreement with several international adopted guidelines for GMO safety analyses (Codex, 2003; AHTEG, 2011; EFSA, 2011). In order to avoid environmental variation, we have performed a field experiment that consisted of several seed lines out of which individual plants were randomly selected from the inner rows in order to minimize possible field effects derived from border effects or heterogeneities in the field area. For the purpose of addressing plant-to-plant variability within our treatment and control plants, we have performed PCA which demonstrated similarities in the protein quantity between different gels. PCA analytical report shows that the first three components explained 46% of the variation. The PCA plot showed a clear separation between the GM and non-GM plants in the first component, which explained 33.7% of the total variation (Figure 2). There was also biological variation in the analysis, explaining 12.6%. These results show that the plant-to-plant variability fall in the range of what is usually accepted for proteomic analysis.

### Proteomic Profile of White Bt-Maize (MON810) and Its Non-GM Counterpart

**Figure 3:**
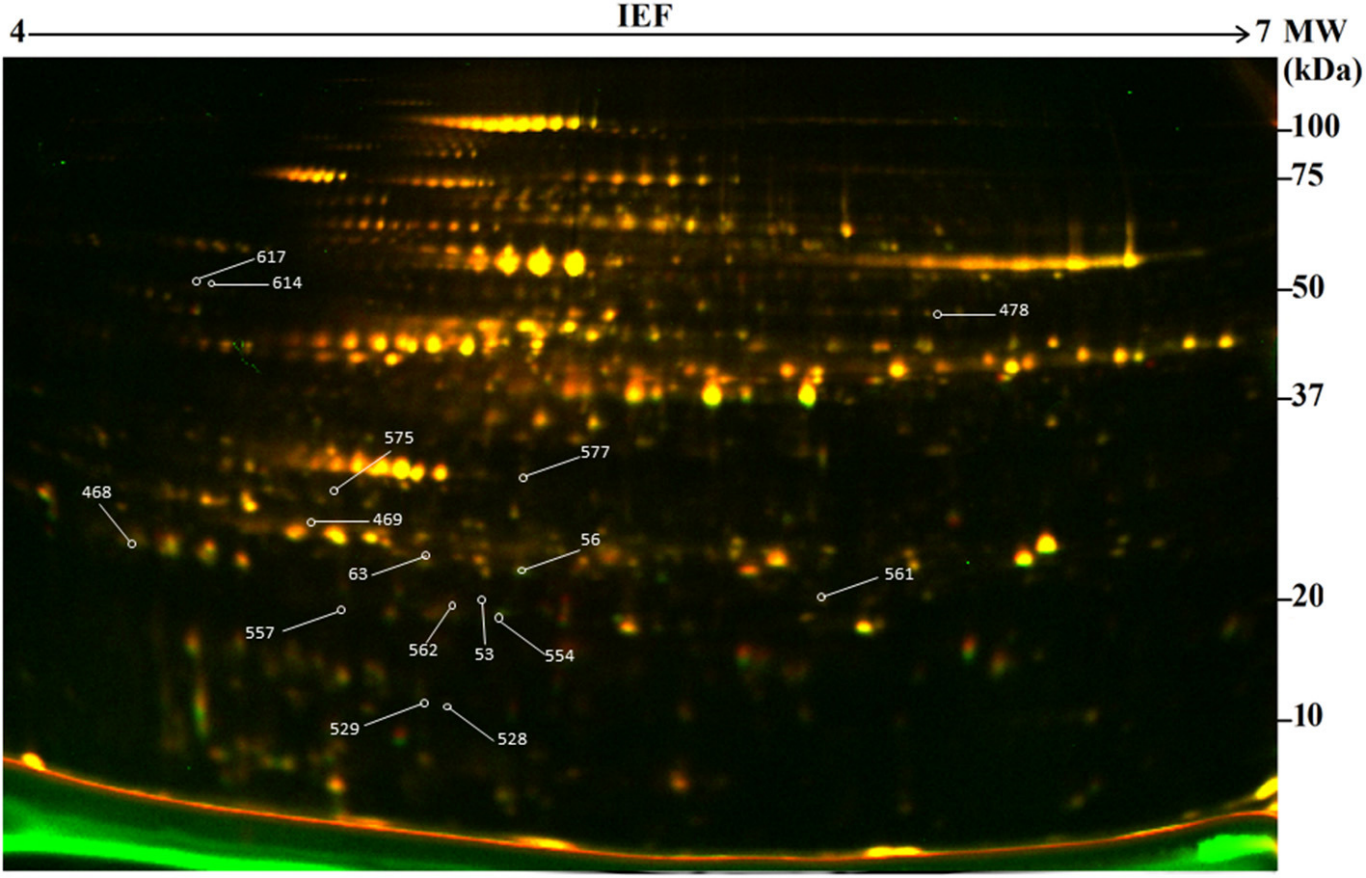
Representative two-dimensional difference gel electrophoresis (2-D DIGE) map of the proteome of genetically modified maize plants (MON810 event) between pH 4 and 7. Delimited spots correspond to differentially expressed proteins selected for mass spectrometry identification. ID of identified proteins from Table 2 is indicated in yellow boxes.

In this study, 2-D DIGE combined with mass spectrometry (MS) was used to develop protein profiles in order to assess new protein products or metabolic differences occurring due to genetic modification resulting from particle bombardment. The proteomic profile of the field-grown white maize MON810 GM variety PAN 6Q-321B, widely grown in South Africa, was compared to its near-isogenic variety PAN 6Q-121; thus mimicking real world agricultural scenarios (Figure 3).

The amounts of total protein extracted were 10.78 ± 1.19 mg/g (dry weight) for the non-GM samples and 11.11 ± 1.57 mg/g (dry weight) for the GM samples. Average numbers of spots on the 2-D DIGE gel were 710 ± 105 (GM) and 820 ± 95 (non-GM). The amount of protein extracted and the number of detected spots from both treatments did not show statistical significant difference (*P* = 0.749 and 0.172; respectively). After manual verification of spots, gels were matched according to hierarchical condition, in which technical repetitions were first compared, followed by biological repetitions comparison and further treatment comparison. Therefore, gels from different treatments were internally matched and only consistent spots were included in the analysis. The average correlation coefficients were 0.91 ± 0.2 for the non-GM sample and 0.92 ± 0.2 for the GM sample with a total number of matched spots of 514 and 669 for the non-GM and GM gels, respectively (Table 1). These results indicate a high degree of sensitivity and reproducibility using the 2D-DIGE/MS approach (Choudhary et al., 2016; de Campos et al., 2020).

**Table 1:** Total protein content, detected spots and matched spots of PAN 6Q-121 non-GM and PAN 6Q-321B GM maize varieties grown under farm conditions in Bloemfontein, South-Africa.

**Variety**	**Total protein content (mg.g^**−1**^ of dry weight)^a^**	**Average n° of spots detected^a^**	**N° of matched spots**	**Exclusive spots (match ID)^b^**	**Differentially expressed spots (match ID; fold of variation)^b^**
PAN 6Q-121 non-GM	10.78 ± 1.19	710 ± 105	514	468; 469; 478	56 (2); 63 (1.5)
PAN 6Q-321B GM	11.11 ± 1.57	820 ± 95	669	528; 529; 554; 557; 561; 562; 563; 575; 577; 614; 617	

a *Values are means of n = 4 gels ± standard deviation*;

b *Spots were considered exclusive or differentially expressed when a Student's t test results were significant (95% confidence interval)*.

*The number of differentially expressed spots from the comparison of both varieties is also presented*.

### Differential Protein Expression Patterns in MON810 Compared to Its Near-Isogenic Line

**Figure 4:**
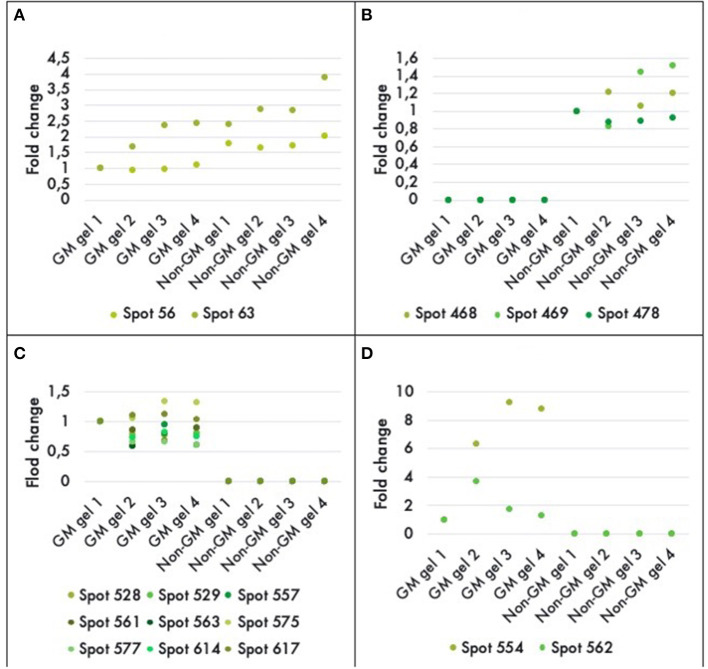
Histograms of fold-differences of proteins found to be significantly different (ANOVA, *P* < 0.05) in leaf samples of genetically modified maize (MON810 event) and non-transgenic near isogenic (PAN 6Q-121) field-grown in Bloemfontein (South Africa; autumn 2009). Sixteen proteins out of an average of 765 spots presented a significant difference in abundance; in which two were down regulated **(A)**, three were repressed **(B)**, and 11 were only expressed in the GM plants **(C,D)**. Spots 554 and 562 had greater values and were, therefore, included in a new histogram. Protein expression levels represent the relative protein expression compared to a reference gel (four technical replicates were used).

A comparison between the GM and non-GM plants revealed a total of 16 different proteins that were either up or down regulated in one of the varieties at a statistically significant level (*P* < 0.05). Eleven out of 16 of the differentially expressed proteins were detectable only in the GM variety. And three proteins were completely repressed in the GM variety while 2 proteins were down-regulated by a factor of 1.5 and 2 (Figure 4). The 16 proteins were successfully identified with C.I.% values <95% using MALDI–TOF-MS/MS analysis (*P* < 0.05) (Table 2).

**Table 2:** MS/MS identification of the differentially expressed proteins in PAN 6Q-321B GM vs. PAN 6Q-121 non-GM maize varieties.

**Protein name**	**Spot n°**	**MW (kDa) theor/ exp**.	**p*I* theor./exp**.	**NCBI accession no**.	**N° of matched peptides**	**Mascot score**	**Fold of variation**	***p-*value**	**Cellular component GO term**	**Genome location (Chromosome number)**	**Molecular function GO term**
Putative cytochrome c oxidase subunit II PS17 [*Pinus strobus*]	56	17/19	9.6/5.2	109892850	3	67	2	0.0002	Chloroplast, membranes and mitochondria	Mitochondrion and 9 (in pine)	Copper ion binding and cytochrome-c oxidase
Adenylate kinase [*Zea mays*]	63	31.2/26	6.8/5.0	195611658	6	277	1.5	0.0073	Chloroplast	3, 6, and 8	Kinase transferase and ATP binding
2-cys peroxiredoxin BAS1 [*Zea mays*]	468	28.3/29	5.8/4.2	195626524	3	152	OFF	0.0001	Chloroplast	4 and 5	Oxidoreductase
2-cys peroxiredoxin BAS1 [*Zea mays*]	469	28.3/23	5.8/5.0	195626524	12	326	OFF	0.0028	Chloroplast	4 and 5	Oxidoreductase
Bifunctional 3-phosphoadenosine 5-phosphosulfate synthetase 2 [*Zea mays*]	478	52.5/47	8.3/6.2	226492878	4	120	OFF	3.07 E-05	Chloroplast stroma, mitochondria and plasma membrane	2	Sulfate adenylyl transferase (ATP)
Hypothetical protein SORBIDRAFT_03g012630 [*Sorghum bicolor*]	528	18.9/12	11/5.2	242057187	2	49	ON	0.0003	No annotation	3 (in sorghum)	Signal transduction
Pathogenesis-related protein 1 [*Zea mays*]	529	17.1/12	5.4/5.1	195615416	2	93	ON	0.0014	Extracellular region	5	Response to biotic stimulus
Chlorophyll a-b binding protein 6A [*Zea mays*]	554	26.5/16	6.2/5.2	226503327	2	164	ON	0.0221	Chloroplast	4 and 5	Metal ion binding
Thylakoid lumenal 19 kDa protein [*Zea mays*]	557	27.4/17	5.5/4.8	226491484	4	147	ON	3.76E-05	Chloroplast	1	Calcium ion binding
Manganese superoxide dismutase (SOD-3) (EC 1.15.1.1) [*Zea mays*]	561	25.6/17	7.1/6.0	168624	6	184	ON	0.0004	Mitochondria	6	Copper ion binding and superoxide dismutase
Thylakoid lumenal 19 kDa protein [*Zea mays*]	562	27.4/17	5.5/5.0	226491484	6	239	ON	0.0258	Chloroplast	1	calcium ion binding
Chlorophyll a-b binding protein 6A [*Zea mays*]	563	26.5/18	6.2/5.1	226503327	2	115	ON	0.0020	Chloroplast	4 and 5	Metal ion binding
14-3-3-like protein A [*Zea mays*]	575	28.7/26	4.9/4.7	226510006	7	348	ON	0.0005	Cytoplasm and nucleus	4	Cis-acting DNA regulation
Lactoylglutathione lyase [*Zea mays*]	577	37.5/27	5.9/5.2	194701526	4	275	ON	0.0018	Cytoplasm	6	Metal ion binding
Chloroplast fructose-1,6-bisphosphatase [*Oryza sativ*a Indica Group]	614	39.2/50	4.7/4.5	165940477	6	227	ON	0.0004	Chloroplast	1; 8 and 9 (in rice)	Phosphoric ester hydrolase
Chloroplast fructose-1,6-bisphosphatase [*Oryza sativa* Indica Group]	617	39.2/50	4.7/4.4	165940477	4	195	ON	2.76E-05	Chloroplast	1, 8, and 9 (in rice)	Phosphoric ester hydrolase

**Figure 5:**
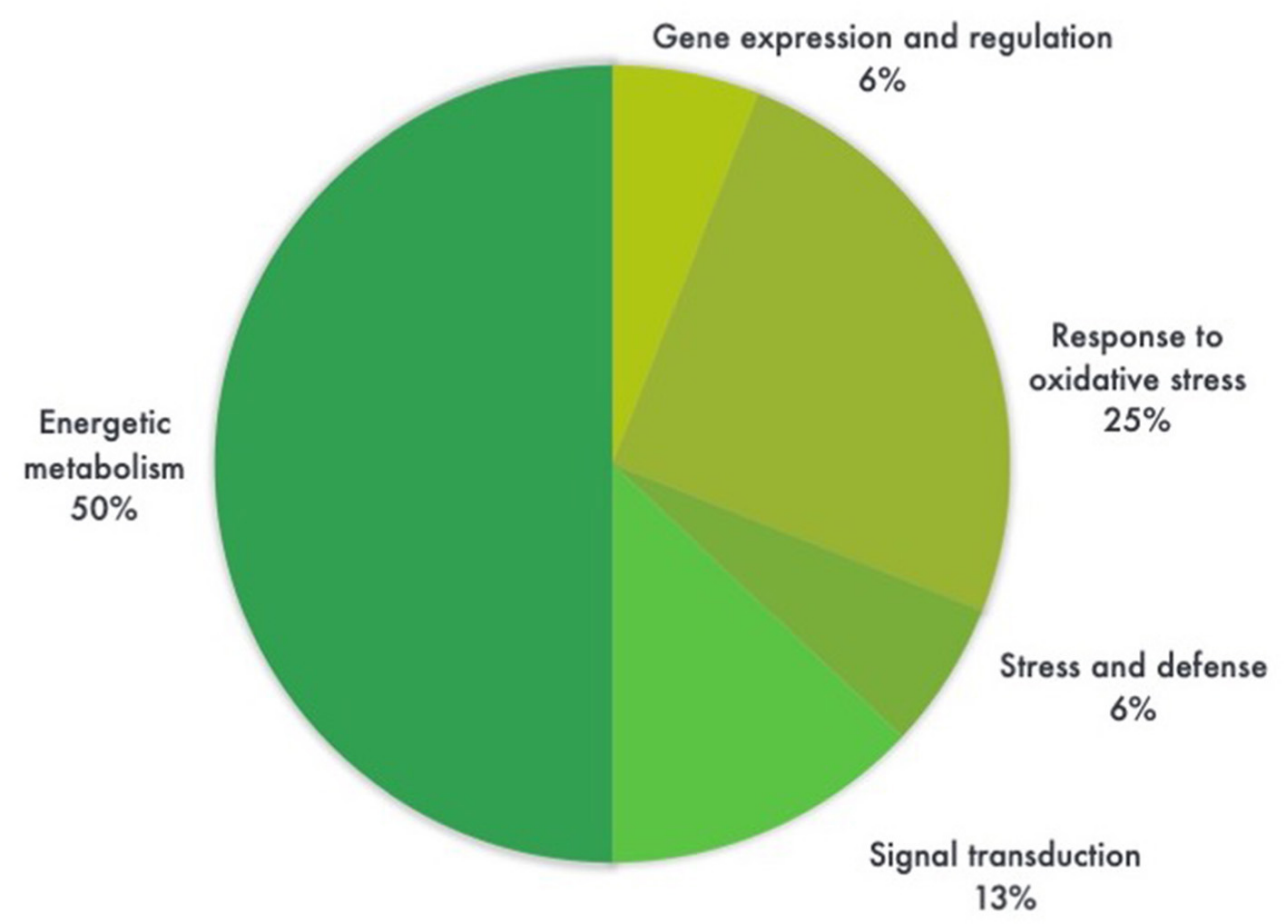
Pie chart distribution of differentially expressed proteins (ANOVA, *P* < 0.05) between leaf samples of genetically modified maize (MON810 event) and non-transgenic isogenic (PAN 6Q-121) based on the function of the proteins. Protein functions were predicted according to Gene Ontology terms. *N* = 16, whereas 6% correspond to a single protein.

Most proteins were specific enzymes closely related to cellular energy homeostasis and reduction-oxidation (redox) metabolism (Figure 5). We found proteins involved in photosynthesis and the synthesis of temporary storage polysaccharide pathways, including adenylate kinase (down-regulated in the GM), bifunctional 3-phosphoadenosine 5-phosphosulfate synthetase (repressed in the GM), thylakoid lumenal 19 kDa protein (expressed in the GM), chlorophyll a-b binding protein 6A (expressed in the GM), all identified in *Zea mays* species; plus chloroplast fructose-1,6-bisphosphatase (expressed in the GM) identified in *Oryza sativa* Indica Group. Likely wise, other energy-related proteins, such as those involved in the photophosphorylation metabolism (ferredoxin–nadp reductase and thylakoid lumenal 19 kda proteinin), were observed in Bt maize grown in field conditions in Brazil (Agapito-Tenfen et al., 2013). Few other studies have also investigated the proteome or the transcriptome profile of GM Bt maize (MON810 event) but the functional information on the identified differential proteins and genes were not available; most likely due to the lack of annotations in databases at the time of publication (Coll et al., 2010; Vaclavik et al., 2013; Balsamo et al., 2015).

We have also observed the cytochrome c oxidase subunit II (over expressed in GM) that is involved in the aerobic respiration, the 2-cys peroxiredoxin BAS1 (repressed in the GM) which catalyzes the transfer of electrons from sulfhydryl residues to peroxides, and the manganese superoxide dismutase enzyme (SOD-3) (expressed in the GM). Superoxide dismutase enzymes (SODs) act as antioxidants and protect cellular components from being oxidized by reactive oxygen species (ROS) (Mittler, 2002; Gill and Tuteja, 2010). ROS can form as a result of various stress conditions, such as drought, injury, pesticides, ozone, plant metabolic activity, nutrient deficiencies, photoinhibition, air and soil temperature, toxic metals, and UV or gamma rays (Smirnoff, 1993; Suzuki et al., 2012). Therefore, these are important enzymes that are closely linked to stress perception and physiological responses to stress.

Disturbances at the redox metabolism of transgenic Bt maize have been previously observed for GM maize lines expressing *cry1Ab/cry2Aj* transgenes (Hao et al., 2017). Field grown Bt maize also showed a different proteomic profile for other antioxidant enzimes, including the 2-cys peroxiredoxin and apx1—cytosolic ascorbate peroxidase (Agapito-Tenfen et al., 2013).

In addition, we found isoforms for five identified proteins (Table 2). These isoforms are photosynthesis-related proteins present in the chloroplast and some with specific functions in phosphoric ester hydrolase activity, calcium ion binding and oxidoreductase activity.

Although protein identity matches were highly confident, we observed differences between theoretical and expected molecular weight and *pI*. Similarly, when analyzing GM maize seed lines, Zolla et al. (2008) found that a number of seed storage proteins (such as globulins and vicilin-like embryo storage proteins) exhibited truncated forms with a molecular mass significantly lower than native ones. Our results also showed lower molecular weight for photosynthesis-related, signal transduction and pathogenesis-related proteins. As for *pI* changes, most of our identified proteins showed lower *pI* than the theoretical values. Considering that we have high resolution gels with little or no smear, and the protein identity match is high confident, the low *pI* values may be evidence of a post-translational modification, cleavage or alternative-splicing event.

Moreover, no transgenic protein products (Cry1Ab) derived from the transgene inserted into MON810 event were revealed in our 2-D DIGE gels. We hypothesized that the extraction buffer at pH 8.0 does not allow Cry1Ab solubilization, which is well-known to be solubilized around pH 11 (Zolla et al., 2008; Balsamo et al., 2011).

Genes are not randomly distributed in the genome and their coordinated expression can be regulated by many factors at virtually any step of gene expression; from transcriptional initiation, to RNA processing, and to the post-translational modification of a protein. Nevertheless, in the case of a transgenic organism, the insertion and expression of a transgene can also be a source of endogenous gene modulation (Latchman, 2005). Similarly, different copies of the introduced gene which integrate into the host chromosomes at different positions are expressed at very different levels, suggesting that gene activity is being influenced by adjacent chromosomal regions (Li et al., 1999). We have included the genome location of each of the differentially expressed protein found in this study to our analysis (Table 2). Interestingly, the genome location of differentially expressed genes varied, thus showing the influence of MON810 transgene integration site, into other genomic locations, likely due to changes in chromatin structure (e.g., heterochromatin) or inserted sequences acting as transcriptional regulation elements (e.g., enhancers, strong promoters) (Weising et al., 1988).

Independent researchers have sequenced flanking regions at both ends and found results that matched to sequences in different chromosomes. Holck et al. (2002) found that the maize endogenous DNA showed high similarity, 88% across the 440 bp next to the 5′ end junction, with the *Zea mays* chromosome 4 22 kD alpha *zein*-gene cluster region (accession number AF105716). And Rosati et al. (2008) found 99% identity of the junction 3′ end with the chromosome 5 BAC clone ZMMBBc0409B05 (accession number AC185641). The latter having 82% identity with *Oryza sativa* locus coding for a putative HECT E3 ubiquitin ligase. These results suggest that the integration of the MON810 vector has probably caused a complex recombination event as the 5′ e 3′ end regions do not correspond to the same genomic locus.

We identified six proteins that were located in chromosomes 4 and 5—the putative location of MON810 insert. Other proteins were found to be located at all maize chromosomes, with the exception of chromosome 7, 10, and circular chloroplast chromosome. Apparently, these genes are not clustered together but dispersed in the maize genome. And their expression does not seem to be controlled by a common regulatory process. These should, however, be further investigated.

### The Allergenic Potential of the Protein Isoform in White Bt-Maize

We found an unique spot (Match ID 529) corresponding to the pathogenesis-related protein class 1 (PR1) was expressed only in the GM variety. Annotations for this protein in the ExPASy Tool related it to a 160 amino acid residues protein with a 17.1 kDa mass and belonging to the Bet v-1family of proteins. The betallergens have a signature represented by a 7-element fingerprint which was derived from an initial alignment of motifs that were drawn from short conserved regions spanning the full alignment length of 45 sequences (Attwood et al., 2003). *Zea mays* PR1 has not been experimentally characterized, but it is 85.7% identical in amino acid sequence to the well-characterized PR1 from *Sorghum bicolor* (Q41298_SORBI).

Epitope search in the allergen database for food safety (http://allergen.nihs.go.jp) using *Z. mays* PR1 as the query, revealed high matches to two known allergens: PR1 from *Asparagus officinalis* and Pru av1 from *Prunus avium. A. officinalis* PR1 was a 51% identity match with *Z. mays* PR1 and *P. avium* AV1 was a 42.4% match (Supplementary Table 1). The Allergen Database for Food Safety is based on a Joint FAO/WHO Expert Consultation on Foods Derived from Biotechnology report which proposed that cross-reactivity between a query protein and a known allergen has to be considered when there is more than 35% identity match in the amino acid sequence of the expressed protein, using a window of either 80 amino acids and a suitable gap penalty or the identity of 6 contiguous amino acids. The epitope search results in relation to PR1 from *A. officinalis*, produced a score of 173, 51% identity and an E-Value = 1e-42. The search result related to the prediction of allergenicity produced a match with Pru av1 from *P. avium* with a score of 481.4, 42.4% identity and an E-Value = 4.8e-22. Spangfort et al. (2003) studied IgE-binding epitope of Bet v-1 from *Betula* sp. and verified that the epitope occupies 10% of the molecular surface area of the protein. These authors found that it is clearly conformational and has a sequential motif around residues 42–52. Our results show a full match with the same sequential motif (43–50 residues) for the Pru av1 data. Further, our peptide sequence obtained from the MS/MS results produced a true match to the epitope region.

The allergenic potential of the Bet v-1 like protein, to which the PR proteins are related, are common pollen and plant food allergens that have been widely described, including their possible isoforms (Reuter et al., 2005). In a recent review, Schenk et al. (2010) show evidence of variation in the immune reaction to different isoforms of Bet v-1 allergens as the allergenicity of pollen from a particular biological source is not determined by the total allergen content alone, but also by the quantities of the different isoforms and their allergenic potential. To the best of our knowledge, there is no investigation on transgenic maize leaf allergens reported in the literature.

A recent study reveals differential reactivity of plasma from two maize allergic subjects against transgenic (MON810) vs. non-transgenic grain protein extracts; however, it was not possible to identify the putative allergens (Fonseca et al., 2012). Although the authors were not able to observe differential expression for the tested allergen genes presented in GM vs. non-GM varieties, these authors have confirmed the reactivity of chitinase and endochitinase A proteins in maize samples, both belonging to the group of the “pathogenesis-related proteins.”

Nonetheless, the potential allergen isoform identified in this study (PR1) shows high homology to the major cherry allergen (Pru av1) and 97 of 101 (96%) patients with birch pollinosis and oral allergy syndrom to cherry had IgE against Pru av 1 (Scheurer et al., 2001). It is, therefore, highly recommended that further studies should be performed in order to investigate the expression of PR1 and its allergenic potential.

Coll et al. (2009) investigated field-grown maize leaves at the vegetative two-leaf (V2) and the tasseling (VT) stages and compared those results to the previous study with the same varieties at V2 stage under *in vitro* conditions by transcriptomics. These authors found four transcripts out of 36 that were differentially expressed between GM (MON810 event) and its conventional counterpart. These were identified as pathogenesis related protein (PR-9), trypsin inhibitor gene, Myb-like protein E1 and one uncharacterized RNA-binding protein. It is interesting to note that PR-9 was overexpressed in the GM plants in all stages and conditions analyzed, but the fold variation was greater for V2 plants grown under field-conditions. PR-9 and PR-10 represent two protein classes, the first being a peroxidase-like and the second a ribonuclease-like protein. Although these proteins participate in different metabolic pathways, both are produced in plants in the event of a pathogen attack. The distinct expression level between GM and non-GM plants reveals that the presence of an insecticide protein (Bt) might challenge plant-pathogen response differentially. Evidence to support this idea has been observed by Coll et al. (2011) study, which has investigated the proteome of GM maize grains grown under field conditions. Coll et al. (2011) found 4 and 6 differentially expressed proteins in two different commercialized maize hybrids in Spain. These authors concluded that the differential expression was variety-specific manner and called attention to the fact that depending on the experimental conditions applied, the analysis concerned a defined window in terms of pI and MW and are restricted to soluble and abundant proteins.

In principle, it is possible that the allergenic potential of GMOs may be increased due to the introduction of potential foreign allergens, to potentially upregulated expression of allergenic components caused by the modification of the wild type organism or to different means of exposure. It is suggested for GMO risk analysis that experimental comparison of the wild-type organism with the whole GMO regarding their potential to elicit reactions in allergic individuals and to induce *de novo* sensitizations should be investigated along with the current evaluation of physiochemical properties and sequence homology with known allergens (Spök et al., 2005).

Therefore, we strongly suggest that further studies should be performed in order to investigate the nature and the allergenic potential of the PR1 protein isoform found in our study.

### Relevance of the Use of Profiling-Techniques in Comparative Risk Assessments and Contributions to the Method Development

Proteomics and the use of bi-dimensional gel electrophoresis have long been tested as analytical tools that can complement existing risk assessment methods. Bi-dimensional gels have the capacity of characterizing and distinguishing varieties and genotypes, identifying possible allergens present in a sample, and detecting possible posttranslational modifications (Zolla et al., 2008).

Other studies have revealed that transgenic plants react differently to environmental conditions as compared to their near isogenic counterparts (Coll et al., 2010; Agapito-Tenfen et al., 2013; Zhao et al., 2015; Hao et al., 2017). In these studies, differences in gene expression, both proteins and metabolites, are not a product of the transgene *per se*. The differently expressed products are affected by the genetic manipulation in which the process of transformation seems to cause insertional or pleiotropic changes in the maize proteome. Several other studies have investigated the proteome of transgenic maize grains due to concerns about human and animal consumption (Zolla et al., 2008; Brandão et al., 2010; Vidal et al., 2015; Liu et al., 2018). It is relevant to mention that, when released into the environment, humans and animals are in contact with the GMO by several different exposure routes which is not always related to feeding grains alone. Humans and animals are exposed, for example, to pollen and herbivores are exposed to leaves through feeding. In addition, at the hazard identification step, any genotypic and phenotypic differences in the GMO should be scrutinized for their potential safety effect. According to the Guidance on Risk Assessment of Living Modified Organisms (AHTEG, 2011), the “exposure assessment” aims to determine whether the receiving environment will be exposed to a living modified organism (LMO) that has the potential to cause adverse effects, taking into consideration the intended transfer, handling and use of the LMO, and the expression level, dose and environmental fate of transgene products.

In our study, field-grown maize was sampled in order to investigate the proteome of maize leaves under real field conditions. Therefore, our approach provides an important insight into environmental risk assessments (ERA) with regards to possible impacts on herbivores and/or other pathogen communities that feed on the transgenic maize leaf.

Current ERA practice of GM maize for food and feed or cultivation purposes in the European Union have been challenged due to the assumption that GM plants consist of two parts that function in a linear additive fashion: the crop and the novel GM transgene product (Dolezel et al., 2011). This assumption is based on the substantial equivalence concept; when no statistically relevant compositional changes are detected, the crop plant is declared as safe and consequently only the added transgene product is subject to testing in the environmental risk assessment. Our study shows that the analysis of a larger and unknown set of proteins (proteomic profile) is useful to comprehensively screen for unintended changes in genetically modified plants.

It is important to note that our findings might be specific to the sample used, especially because these proteins are highly environmentally dependent. Therefore, case-by-case studies should be performed in order to provide reliable results for a specific type of risk assessment. The detection of changes in protein profiles does not present a safety issue *per se*; however, our data shows that the GM variety is not substantially equivalent to its non-transgenic near-isogenic variety. Therefore, further studies should be conducted in order to address the biological relevance and the potential risks of such changes.

In the light of speed of development of new GMOs, new tools such as omics are needed to enable a comprehensive risk assessment of more complex transgenic modifications and traits. Therefore, regulatory agencies may take into account proteomic and other omics studies among the required ones.

## Conclusions

In conclusion, our results showed that GM Bt maize grown in South Africa were clustered together and distant from on-GM genotypes analyzed by PCA which explained cerca 34% of the variation in the dataset. In addition, we obtained evidence of possible synergistic and antagonistic interactions following Bt transgene insertion into the GM maize genome. This conclusion is based on the observation of several metabolic processes that were disturbed in the GM samples alone. These proteins were mainly assigned to the energy/carbohydrate metabolism and also found in previous studies. In addition, a potential allergenic protein PR1 was also observed in the GM Bt samples, in which the epitope has been sequenced by MS/MS. Such observations indicate that the genome changes in Bt GM maize may influence the overall gene expression in ways that may have relevance for hazard identification assessments. Therefore, we concluded that this GM maize variety is not substantially equivalent to its near-isogenic non-transgenic counterpart.

## Data Availability Statement

The datasets presented in this study can be found in online repositories. The names of the repository/repositories and accession number(s) can be found in the article/Supplementary Material.

## Author Contributions

O-GW and SZ: conceptualization, formal analysis, and methodology. SZ: data curation, writing—original draft, and validation. O-GW, MG, and RN: funding acquisition and resources. O-GW, SZ, MG, and RN: investigation. O-GW: project administration and supervision. O-GW, MG, RN, and SZ: writing—review and editing. All authors have read and agreed to the published version of the manuscript.

## Conflict of Interest

The authors declare that the research was conducted in the absence of any commercial or financial relationships that could be construed as a potential conflict of interest.
