# Supplementary material for: Untargeted Proteomics-Based Approach to Investigate Unintended Changes in Genetically Modified Maize for Environmental Risk Assessment Purpose
Source: Front Toxicol. 2021 Jun 22;3:655968. doi: 10.3389/ftox.2021.655968 (PMC8915820; doi:10.3389/ftox.2021.655968)
Supplement: Supplementary file 1 [file Table_1.docx]

Table 1. Alignment of amino acid sequence of PR1 and related proteins from different plants according to database search. The predicted maize PR1 sequence is compared with the primary structures of the sorghum PR10 protein (Alexandrov et al., 2009); cherry Pru av1 protein (Reuter et al., 2005) and asparagus AoPR1 protein (Warner et al., 1992).

| Database search | Protein name (organism) | Sequence similarity | Score | E-value |
| --- | --- | --- | --- | --- |
| Expasy^1^ | Pathogenesis-related protein **– PR10 (*Sorghum bicolor*)** | MASVNSWTLEIASPVAPQRLFRAAVMDWHTLA-**PKVASHVV**  **ASAQPVEGDGGVGSVR**QFNFTSVMPFSFMKERLEFLDADKCECKNTLIEGGGIGVAIETATSHIK**VEPAAGGGSVVK**VESTYKLLPGVEVKDEIAKAKESVTAIFKGAEAYLVANPDAYN | 647 | - |
| Allergenicity prediction^2^ | Major cherry allergen - Pru av1 (*Prunus avium*) | MASVNSWTLEIASPVAPQRLFRAAVMDWHTLA**PKVASHVVASAQPVEGDGGVGSVR**QFNFTSVMPFSFMKERLEFLDADKCECKNTLIEGGGIGVAIETATSHIK**VEPAAGGGSVVK**VESTYKLLPGVEVKDEIAKA-KESVTAIFKGAEAYLVANPDAYN | 481.4 | 4.8 e^-22^ |
| Epitope (FAO/WHO based)^2^ | Pathogenesis-related protein 1 - AoPR1 (*Asparagus officinalis*) | MASVNSWTLEIASPVAPQRLF**RAAVMDWHTLAPKVASHVVASAQPVEGDGGVGSVR**QFNFTSVMPFSFMKERLEFLDADKC ECKNTLIEGGGIGVAIETATSHIK**VEPAAGGGSVVK**VESTYKLLPGVEVKDEIAKAKESVTAIFKGAEAYLVANPDAYN | 173 | 1.0 e^-42^ |

^1^Swiss Institute for Bioinformatics (http://expasy.org)

^2^Allergen Database for Food Safety (http:// allergen.nihs.go.jp/ADFS)

True matches are highlighted in gray. IgE-binding epitope of Bet v 1 from *Betula* sp. is underligned (Spangfort et al., 2003). Bold letters indicate peptide sequence obtained from MS/MS results.
